# Awareness, expectation and satisfaction towards ward pharmacy services among patients in medical wards: a multi-centre study in Perak, Malaysia

**DOI:** 10.1186/s12913-021-07185-7

**Published:** 2021-10-29

**Authors:** Chew Beng Ng, Chee Tao Chang, Su Yin Ong, Maslinatasha Mahmud, Lay Chin Lee, Wei Yee Chew, Normi Hamdan, Ros Sakinah Kamaludin, Kah Shuen Thong, Shea Jiun Choo

**Affiliations:** 1 Pharmacy Department, Hospital Taiping, Ministry of Health Malaysia, Taiping, Malaysia; 2 Clinical Research Centre, Hospital Raja Permaisuri Bainun, Ministry of Health Malaysia, Ipoh, Malaysia; 3 Perak Pharmaceutical Services Division, Ministry of Health Malaysia, Ipoh, Malaysia; 4 Pharmacy Department, Hospital Parit Buntar, Ministry of Health Malaysia, Parit Buntar, Malaysia; 5 Pharmacy Department, Hospital Teluk Intan, Ministry of Health Malaysia, Teluk Intan, Malaysia; 6 Pharmacy Department, Hospital Selama, Ministry of Health Malaysia, Selama, Malaysia; 7 Pharmacy Department, Hospital Seri Manjung, Ministry of Health Malaysia, Seri Manjung, Malaysia; 8 Pharmacy Department, Hospital Slim River, Ministry of Health Malaysia, Slim River, Malaysia; 9 Pharmacy Department, Hospital Raja Permaisuri Bainun, Ministry of Health Malaysia, Ipoh, Malaysia

**Keywords:** Patient, Awareness, Expectation, Satisfaction, Ward pharmacy, Malaysia

## Abstract

**Background:**

Patient’s awareness and satisfaction towards ward pharmacy services may influence perception towards effectiveness and safety of drugs, affecting medication adherence and clinical outcome. Nevertheless, studies on local ward pharmacy services were lacking. This study evaluated awareness, expectation and satisfaction of ward pharmacy services among patients in medical wards and determined their association with demographic characteristics.

**Methods:**

This was a cross-sectional study using self-administered questionnaire conducted in medical wards of fourteen Perak state public hospitals from September to October 2020. In-patients aged ≥18 years old were included. The validated questionnaire had four domains. The student’s t-test, one-way analysis of variance (ANOVA) and multiple linear regression were was employed to evaluate the association between patients’ demographic characteristics with their awareness, expectation and satisfaction towards ward pharmacy services.

**Results:**

467 patients agreed to participate (response rate = 83.8%) but only 441 were analysed. The mean age of the patients was 54.9 years. Majority was male (56.2%), Malay (77.3%), with secondary education (62.9%), rural resident (57.1%) and reported good medication adherence (61.6%). The mean awareness score was 49.6 out of 60. Patients were least aware about drug-drug interaction (3.85 ± 1.15) and proper storage of medications (3.98 ± 1.06). Elderly patients (β = − 2.82, *P* < 0.001) obtained lower awareness score. Patients with tertiary education (β = 3.87, *P* = 0.001), rural residents (β = 3.65, *P* < 0.001) and with good medication adherence (β = 2.55, *P* = 0.002) had higher awareness score. The mean expectation score was 44.0 out of 50. The patients had higher expectation to encounter a polite ward pharmacist (4.51 ± 0.56). Patients with tertiary education (β = 1.86, *P* = 0.024), rural residents (β = 1.79, *P* = 0.001) and with good medication adherence (β = 1.48, *P* = 0.006) demonstrated higher expectation. The mean satisfaction score was 43.6 out of 50. The patients had high satisfaction in language used (4.45 ± 0.57) and level of knowledge demonstrated (4.41 ± 0.62) by the ward pharmacists. Patients with tertiary education (β = 2.16, *P* = 0.009), rural residents (β = 1.82, *P* = 0.001) and with good medication adherence (β = 1.44, *P* = 0.009) demonstrated higher satisfaction, while elderly patients (β = − 1.17, *P* = 0.031) had lower satisfaction towards ward pharmacy services.

**Conclusion:**

There was a high level of awareness, expectation and satisfaction towards ward pharmacy services in public hospitals of Perak, Malaysia.

## Background

Traditionally, pharmacists’ professional job scope focused on the manufacture and supply of medicines, with minimal interactions with other healthcare professionals and patients [1]. The increasing complexity of medication regimen and high occurrence of medication errors had facilitated the change of pharmacy services to a more patient-orientated role [2]. Participation of ward pharmacists in patient care was found to be beneficial in improving patients’ clinical outcome, reducing hospitalization and adverse drug events [3, 4]. Furthermore, ward pharmacists’ involvement as part of the multidisciplinary team is a cost- effective measure, associated with improved patient safety and satisfaction [5].

In year 2004, ward pharmacy service was initiated in the Malaysian government hospitals. Ward pharmacists were trained in pharmacotherapy, partnering with other health care team to ensure optimal treatment outcomes [6]. Within this context, ward pharmacists were routinely involved in patients’ medications reconciliation, adherence assessment, prescriptions screening, bedside counseling and dispensing [7]. These continuums of processes required proactive communication between pharmacists with their patients.

Conventionally, patient satisfaction is an essential component used to evaluate the quality of patient care [8]. Better understanding of pharmacist’s roles and responsibilities is important to enhance pharmacist-patient relationship and patients’ satisfaction towards pharmacy services [9]. Previous studies reported varied degree of dissatisfaction towards the services provided by hospital pharmacists, which suggested the need for further quality improvement [10–13].

Evaluation of patients’ satisfaction toward ward pharmacy services was important to identify the gaps and factors influencing it, which could provide timely feedback for health services improvement. Additionally, patients’ awareness and expectation towards ward pharmacy services might affect their perception towards the effectiveness and safety of drugs, indirectly influencing their acceptability of medication, adherence and clinical outcome [5, 14]. Notably, the presence of clinical pharmacists may reduce length-of-stay by 1.74 days and increased patient’s satisfaction by 1.49 times [5].

Whilst there were several studies which reported the awareness and satisfaction of patients towards pharmacy services, local reports in this aspect are lacking [14, 15]. Therefore, this study aimed to evaluate the awareness, expectation and satisfaction of hospitalized patients towards medical ward pharmacy services in the public hospitals of Perak state, Malaysia.

## Methods

This was a multicentre cross-sectional study conducted in the medical wards of 14 public hospitals in the Perak state of Malaysia from September 2020 to October 2020. Patients aged 18 years old and above were eligible for this study, while patients who could not understand Malay or English language were excluded.

One male and one female medical ward were selected for data collection from each hospital. The number of samples required from each hospital was stratified using the total number of admissions in the selected medical wards. Subsequently, systematic random sampling was employed using the patients’ registration number, in which the patients’ registration number ending with odd numbers who fulfilled the inclusion criteria were sampled.

The minimum sample size for this study was estimated based on the primary outcome the prevalence of patients with good satisfaction towards ward pharmacy practices. According to a previous study, 51.9% of the patients were satisfied with the ward pharmacy services [16]. Sample size was calculated using Raosoft Sample Size Calculator. (http://www.raosoft.com/samplesize.html). Based on a 95% confidence interval, ±5% precision with an infinite population size, a minimum sample size of 377 was required.

The self-administered questionnaire was developed by the investigators based on previously validated surveys [13, 14, 16]. Questionnaire was initially developed in the English version, and subsequently translated into the Malay language. The questionnaires consisted of four sections, i.e. Section I: demographics background of patients; Section II: awareness towards ward pharmacy services (12 item); Section III: expectation towards ward pharmacy services (10 item); Section IV: satisfaction towards ward pharmacy services (10 item). Respondents’ awareness, expectation and satisfaction towards ward pharmacy services were evaluated by using a 5-point Likert-scale (1 = strongly disagree, 2 = disagree, 3 = neutral, 4 = agree and 5 = strongly agree). Patients’ medication adherence at home was measured at the point of admission using the validated MyMAAT score, in which a score of equal or more than 50 was considered as good adherence.

The questionnaire underwent face validation and content validation by two experts in the pharmacy field, and subsequently underwent pre-test on 10 respondents to examine clarity. A pilot test was conducted among 15 respondents to evaluate its reliability and validity. The reliability of the three primary domains was demonstrated through satisfactory Cronbach’s alpha value (α > 0.7): section II (awareness, 0.844); section III (expectation, 0.774); section IV (satisfaction, 0.969).

The survey form was distributed to the patients for self-administration before they discharged from ward. Respondents returned the completed questionnaires to the designated pharmacists before discharge. To avoid social desirability bias, a trained pharmacist in the same hospital but not working in the selected ward was assigned as the data collector. Before administration of questionnaire, the purpose of the study was explained in detail and written informed consent was obtained from the patients before their participation. This study was registered with the Malaysia National Medical Research Registry (NMRR) and ethics approval was obtained from the Medical Research & Ethics Committee (MREC), Ministry of Health Malaysia before its commencement.

### Data analysis

Data was initially entered into the Microsoft Excel 2013 (Microsoft Corporation, Redmond, Washington, United States), and subsequently coded and analyzed using IBM SPSS statistical software version 20.0 (SPSS Inc., Chicago, Illinois). Normality of the awareness, expectation and satisfaction scores were determined by skewness and kurtosis. The values for skewness and kurtosis between − 1.96 and + 1.96 indicates normal distribution. Patients’ demographic characteristics, awareness, expectation and satisfaction scores were descriptively analyzed and reported. In the awareness and expectation domains, each “strongly disagree” response was given 1 point, and each “strongly agree” response was given 5 points. In the satisfaction domain, each very dissatisfied response was given 1 point, while each very satisfied item was given 5 points. The minimum and maximum score range for each domain was as follow: awareness on ward pharmacy services (12–60); expectation on ward pharmacy services (10–50); satisfaction on ward pharmacy services (10–50). The mean score for each item was calculated by averaging the scores with the total number of respondents.

The student’s t-test and one-way analysis of variance (ANOVA) were used to analyze the differences of mean scores in the awareness, expectation and satisfaction domains across different demographic characteristics. Logistic regression was performed to evaluate the influence of awareness scores, expectation scores and satisfaction scores towards adherence. Multiple linear regression was employed to evaluate the association between patients’ demographic characteristics with their awareness, expectation and satisfaction towards ward pharmacy services, elucidated by adjusted beta coefficients (β), standard errors, t-value and *P*-value. We used Pearson’s correlation to determine the relationship between awareness scores, expectation scores and satisfaction scores. Statistically significant level was set at 5%.

A post-hoc reliability and factor analysis was further performed (*n* = 441) for questionnaire validation purposes. The 3 domains obtained good reliability scores: awareness (Cronbach α: 0.909), expectation (Cronbach α: 0.913), satisfaction (Cronbach α: 0.950). Factor analysis was used to determine the appropriate number of domains and whether the items fit the specific construct. The construct validity was assessed using exploratory factor analysis. The questionnaire’s items were extracted using principal component analysis and varimax rotation, and those domains with eigenvalues more than one were retained. Items with factor loadings of more than 0.40 were considered as good fit. Kaiser-Meyer-Olin (KMO) measure of sampling adequacy value of 0.945 indicates sampling sufficiency, while the Barlett’s Test of the Sphericity (*P* < 0.001) indicated that the items were apt for factor analysis. The factor loadings range were found to be satisfactory: awareness (0.470–0.757), expectation (0.575–0.783) and satisfaction (0.688–0.781). This indicates that the items were distinctively related to each other and relevant to the specific domain.

## Results

Out of 557 invited patients, 467 agreed to participate (response rate: 83.8%). A total of 441 respondents were included in final analysis as 26 respondents had missing primary outcomes. The mean age of the patients was 54.9 ± 15.1 years. Majority of the patients were male (248, 56.2%), Malay (340, 77.3%), with secondary education (275, 62.9%), had known medical illness (414, 93.9%), earning a low household income (354, 82.3%) and had reported good adherence towards their medications (257, 61.6%) (Table 1). The scores for the three domains were normally distributed: awareness scores (skewness: − 0.685, kurtosis: 0.496), expectation scores (skewness: − 0.251, kurtosis: − 1.056) and satisfaction scores (skewness: − 0.272, kurtosis: − 0.649).

**Table 1:** Demographic characteristics of patients (*n* = 441)

Characteristics	Frequency	Percentage
**Age in Mean (standard deviation)**	54.9 (15.1)	Range: 18–90
< 60 years	249	56.5
60 years and above	192	43.5
**Admission events in the previous one year (Mean, standard deviation)**	1.54 (1.88)	Range: 0–14
Nil	135	30.6
1–2 (annually or half annually)	222	50.3
3–4 (quarterly)	55	12.5
5 or more (frequent admission)	29	6.6
**Gender**		
Male	248	56.2
Female	193	43.8
**Ethnicity**^**a**^		
Malay	340	77.3
Chinese	40	9.1
Indian	53	12.0
Others	7	1.6
**Education**^**b**^		
No formal education	3	0.7
Primary	99	22.7
Secondary	275	62.9
Tertiary	60	13.7
**Occupation**		
Private	151	34.2
Government	41	9.3
Retiree	99	22.4
Student	10	2.3
Housewife	110	24.9
Unemployed	30	6.8
**Known medical illness**^**c**^		
Yes	414	93.9
No	27	6.1
**Area of residence**^**d**^		
Urban	186	42.9
Rural	248	57.1
**Household income**^**e**^		
Below RM 4360	354	82.3
RM 4360 to RM 9619	69	16.0
More than RM 9619	7	1.6
*Medication adherence scores^**f**^		
Good	257	61.6
Moderate and poor	133	31.9
Not applicable (NKMI)	27	6.5

^a^Missing (*n* = 1) ^b^Missing (*n* = 4) ^c^ Missing (*n* = 2) ^d^Missing (*n* = 7) ^e^Missing (*n* = 11) ^f^Missing (*n* = 24)

*Medication adherence was measured using MyMAAT score, a validated assessment tool used in public hospitals of Malaysia, with score ≥ 50 considered good adherence

Out of a maximum score of 60, the mean awareness score was 49.6 ± 7.95, ranged from 23 to 60. Among all the ward pharmacy services, the patients were least aware about drug-drug interaction (3.85 ± 1.15), proper storage of medications (3.98 ± 1.06), monitoring of patients’ response to drugs (4.00 ± 1.00), explanation on medication side effects (4.01 ± 1.05) and information on changes of medications (4.07 ± 0.98) (Table 2). Elderly population (vs. non-elderly, β = − 2.82, *P* < 0.001) obtained a lower awareness score. Patients with tertiary education (vs. primary education or below, β = 3.87, *P* = 0.001), from the rural areas (vs. urban areas, β = 3.65, *P* < 0.001) and with good adherence towards medications (vs. poor or moderate adherence, β = 2.55, *P* = 0.002) had higher awareness score (Table 3). There was a positive and significant association between awareness scores (OR: 1.028, CI: 1.002–1.056, *P* = 0.034), expectation scores (OR:1.054, CI: 1.011–1.099, *P* = 0.012), satisfaction scores (OR: 1.042, CI: 1.001–1.084, *P* = 0.043) with the level of adherence (Table 4).

**Table 2:** Awareness towards ward pharmacy services (*n* = 441)

No.	Statement	Strongly disagree	Disagree	Neutral	Agree	Strongly agree	Mean (SD)
1.	There are ward pharmacists present in the ward besides doctors and nurses.	6 (1.4)	21 (4.8)	26 (5.9)	213 (48.3)	175 (39.7)	4.21 (0.85)
2.	I was informed by the ward pharmacists to bring my medications upon each admission.	11 (2.5)	36 (8.2)	20 (4.5)	213 (48.3)	161 (36.5)	4.10 (0.97)
3.	Ward pharmacists interviewed me regarding the medications I took at home.	6 (1.4)	18 (4.1)	21 (4.8)	213 (48.3)	183 (41.5)	4.24 (0.83)
4.	Ward pharmacists informed me regarding what the medications are used for.	7 (1.6)	14 (3.2)	14 (3.2)	200 (45.4)	206 (46.7)	4.34 (0.81)
5.	Ward pharmacists explained to me how to take the medications.	5 (1.1)	7 (1.6)	11 (2.5)	207 (46.9)	211 (47.8)	4.39 (0.72)
6.	Ward pharmacists monitor my response to drugs.	12 (2.7)	32 (7.3)	54 (12.2)	189 (42.9)	154 (34.9)	4.00 (1.00)
7.	Ward pharmacists informed me on the date to come back for medication refill.	4 (0.9)	16 (3.6)	30 (6.8)	214 (48.5)	177 (40.1)	4.22 (0.81)
8.	Ward pharmacists informed me on changes of medications before admission and after discharge.	6 (1.4)	44 (10.0)	31 (7.0)	189 (42.9)	171 (38.8)	4.07 (0.98)
9.	Ward pharmacists explained to me about the medication side effects.	15 (3.4)	43 (9.8)	31 (7.0)	190 (43.1)	162 (36.7)	4.01 (1.05)
10.	Ward pharmacists explained to me about drug-drug and drug-food interaction.	21 (4.8)	57 (12.9)	42 (9.5)	170 (38.5)	151 (34.2)	3.85 (1.15)
11.	Ward pharmacists informed me about proper storage of medications.	21 (4.8)	30 (6.8)	35 (7.9)	202 (45.8)	153 (34.7)	3.98 (1.06)
12.	Ward pharmacists dispense medications to me during discharge.	7 (1.6)	19 (4.3)	23 (5.2)	201 (45.6)	191 (43.3)	4.25 (0.85)

**Table 3:** Multiple linear regression for significant factors associated with awareness, expectation and satisfaction (*n* = 441)

Variable	Coefficient (β)	Standard error	T	P
**Awareness on ward pharmacy services**	***R***^***2***^ **= 0.156**			
Elderly (vs. Non elderly)	−2.82	0.79	−3.548	< 0.001
Tertiary (vs. Primary or below)	3.87	1.19	3.242	0.001
Rural (vs. Urban)	3.65	0.78	4.705	< 0.001
Good adherence (vs. poor or moderate adherence)	2.55	0.80	3.186	0.002
**Expectation on ward pharmacy services**	***R***^***2***^ **= 0.069**			
Tertiary (vs. Primary or below)	1.86	0.82	2.267	0.024
Rural (vs. Urban)	1.79	0.53	3.409	0.001
Good adherence (vs. poor or moderate adherence)	1.48	0.54	2.754	0.006
**Satisfaction on ward pharmacy services**	***R***^***2***^ **= 0.080**			
Elderly (vs. non elderly)	−1.17	0.54	−2.170	0.031
Tertiary (vs. Primary or below)	2.16	0.82	2.642	0.009
Rural (vs. Urban)	1.82	0.53	3.422	0.001
Good adherence (vs. poor or moderate adherence)	1.44	0.55	2.614	0.009

Notes: Backward multiple linear regression analysis. Multicollinearity and interaction term were checked and not found (tolerance < 1.00 and VIF < 10). Linear relationship between the independent and outcome variable was checked using scatter plot of residuals. Normality of response variable was checked using histogram and box-plot of residuals. Model assumptions are fulfilled

**Table 4:** Influence of awareness, expectation, satisfaction scores towards adherence

Variables	Nagelkerke R Square	Coefficient (β	(Standard error)	Odds ratio (95% confidence interval)	*p*-value
**Awareness scores**	*R*^*2*^ = 0.016	0.028	0.013	1.028 (1.002–1.056)	0.034
**Expectation scores**	*R*^*2*^ = 0.022	0.053	0.021	1.054 (1.011–1.099)	0.012
**Satisfaction scores**	*R*^*2*^ = 0.015	0.041	0.020	1.042 (1.001–1.084)	0.043

In term of expectation, the mean expectation score was 44.0 ± 5.14 out of a maximum of 50, ranged from 30 to 50. Ward pharmacists who were polite (4.51 ± 0.56) and used understandable language (4.49 ± 0.63) were highly expected among the patients. Meanwhile, a large proportion of the patients expected explanation on the function of medication (4.50 ± 0.57) and prescription completeness checking (4.49 ± 0.63) (Table 5). Patients with tertiary education (vs. primary education or below, β = 1.86, *P* = 0.024), staying in the rural region (vs. urban, β = 1.79, *P* = 0.001) and with good adherence towards medication (vs. poor or moderate adherence, β = 1.48, *P* = 0.006) demonstrated higher expectation (Table 3).

**Table 5:** Expectation towards ward pharmacy services (*n* = 441)

No.	Statement	Strongly disagree	Disagree	Neutral	Agree	Strongly agree	Mean (SD)
1.	I expect the ward pharmacists to check my prescription for completeness.	3 (0.7)	2 (0.5)	8 (1.8)	191 (43.3)	237 (53.7)	4.49 (0.63)
2.	I expect ward pharmacists to use language that is easily understandable.	2 (0.5)	5 (1.1)	6 (1.4)	190 (43.1)	238 (54.0)	4.49 (0.63)
3.	I expect ward pharmacists to ask me if I have concerns about my medications.	1 (0.2)	6 (1.4)	17 (3.9)	214 (48.5)	203 (46.0)	4.39 (0.65)
4.	I expect the ward pharmacists to explain how each of my medications are supposed to help me.	1 (0.2)	0 (0.0)	10 (2.3)	198 (44.9)	232 (52.6)	4.50 (0.57)
5.	I expect the ward pharmacists to ask how well my medical conditions are controlled.	2 (0.5)	8 (1.8)	31 (7.0)	192 (43.5)	208 (47.2)	4.35 (0.73)
6.	I expect the ward pharmacists to advise me on lifestyle modification.	2 (0.5)	14 (3.2)	34 (7.7)	189 (42.9)	202 (45.8)	4.30 (0.78)
7.	I expect the ward pharmacists to involve me in decision making of my medication.	3 (0.7)	14 (3.2)	28 (6.3)	198 (44.9)	198 (44.9)	4.30 (0.78)
8.	I expect the ward pharmacists to be pleasant and courteous.	0 (0.0)	0 (0.0)	15 (3.4)	184 (41.7)	242 (54.9)	4.51 (0.56)
9.	I expect reasonable privacy during discussion with the ward pharmacists.	9 (2.0)	3 (0.7)	34 (7.7)	202 (45.8)	193 (43.8)	4.29 (0.80)
10.	I expect the ward pharmacists to offer me a choice of information sources relevant to my health problem.	1 (0.2)	4 (0.9)	26 (5.9)	205 (46.5)	205 (46.5)	4.38 (0.66)

The mean satisfaction score was 43.6 ± 5.34 out of 50 and ranged from 26 to 50. The patients had high satisfaction in the language used by the ward pharmacists when discussing drug-related matters (4.45 ± 0.57), level of knowledge that ward pharmacists demonstrated in drug-related issues (4.41 ± 0.62) and response of the ward pharmacists towards drug-related questions (4.38 ± 0.61) (Table 6). Patients with tertiary education (vs. primary or below, β = 2.16, *P* = 0.009), staying in the rural region (vs. urban region, β = 1.82, *P* = 0.001) and with good adherence towards medication (vs. poor or moderate adherence, β = 1.44, *P* = 0.009) demonstrated higher satisfaction, while elderly patients (vs. non-elderly, β = − 1.17, *P* = 0.031) had lower satisfaction towards ward pharmacy services (Table 3).

**Table 6:** Satisfaction of patients towards pharmacy services (*n* = 441)

No.	Statement	Very dissatisfied	Dissatisfied	Neutral	Satisfied	Very satisfied	Mean (SD)
1.	Type and amount of information discussed by the ward pharmacists on drug related matters.	0 (0.0)	2 (0.5)	27 (6.1)	236 (53.5)	176 (39.9)	4.33 (0.61)
2.	Questions asked by the ward pharmacists before dispensing medications like any history of previous drug allergy, disease details, etc.	0 (0.0)	7 (1.6)	26 (5.9)	217 (49.2)	191 (43.3)	4.34 (0.66)
3.	Privacy given by ward pharmacists while discussing with patients and dispensing medications.	0 (0.0)	3 (0.7)	22 (5.0)	240 (54.4)	176 (39.9)	4.34 (0.60)
4.	Level of knowledge that ward pharmacists demonstrate in drug related issues.	1 (0.2)	2 (0.5)	20 (4.5)	208 (47.2)	210 (47.6)	4.41 (0.62)
5.	Response of the ward pharmacists towards questions related to drugs.	0 (0.0)	2 (0.5)	25 (5.7)	217 (49.2)	197 (44.7)	4.38 (0.61)
6.	Language used by the ward pharmacists in discussing drug related matters.	0 (0.0)	1 (0.2)	14 (3.2)	213 (48.3)	213 (48.3)	4.45 (0.57)
7.	Amount of time spent by the ward pharmacists with me.	2 (0.5)	3 (0.7)	37 (8.4)	228 (51.7)	171 (38.8)	4.28 (0.68)
8.	My relationship with the ward pharmacists.	3 (0.7)	3 (0.7)	44 (10.0)	201 (45.6)	190 (43.1)	4.30 (0.73)
9.	Information on disease and other health issues.	2 (0.5)	4 (0.9)	34 (7.7)	225 (51.0)	176 (39.9)	4.29 (0.69)
10.	Ward pharmacist services overall.	2 (0.5)	0 (0.0)	24 (5.4)	194 (44.0)	221 (50.1)	4.43 (0.64)

Among all the sociodemographic variables, there was no difference observed across subjects with different status of underlying diseases and household income (Table 7). The awareness scores had a positive, moderate, significant correlation with the expectation scores (*r* = 0.462, *p* < 0.001) and satisfaction scores (*r* = 0.640, *p* < 0.001). The expectation scores had a positive, moderate, significant correlation with the satisfaction scores (*r* = 0.563, *p* < 0.001) (Table 8).

**Table 7:** Comparison of demographic characteristics with awareness, expectation and satisfaction scores (*n* = 441)

Variable	Awareness		Expectation		Satisfaction	
	**Mean (SD)**	*p*-value	**Mean (SD)**	*p*-value	**Mean (SD)**	*p*-value
**Age**						
< 60 years	51.1 (7.44)	< 0.001	44.4 (5.25)	0.098	44.1 (5.20)	0.009
60 years and above	47.7 (8.19)		43.5 (4.97)		42.8 (5.43)	
**Admission events**						
Nil	49.8 (7.90)	0.103	44.1 (5.35)	0.408	44.1 (5.29)	0.308
1–2	48.9 (8.18)		43.7 (5.05)		43.1 (5.33)	
3–4	51.5 (6.75)		44.5 (5.22)		43.8 (5.40)	
5 or more	51.1 (7.97)		45.2 (4.63)		44.0 (5.46)	
**Gender**						
Male	49.3 (8.12)	0.386	44.0 (5.14)	0.875	43.4 (5.59)	0.456
Female	50.0 (7.73)		43.9 (5.15)		43.8 (4.99)	
**Ethnicity**						
Malay	50.2 (7.46)	0.004	43.9 (5.05)	0.775	43.7 (5.03)	0.770
Chinese	45.4 (10.26)		44.6 (5.72)		43.3 (6.73)	
Indian	49.8 (8.45)		43.9 (5.23)		43.1 (5.67)	
Others	47.9 (6.67)		42.4 (6.27)		42.3 (8.81)	
**Education**						
Primary or below	48.3 (8.33)	0.002	43.5 (5.46)	0.123	43.6 (5.05)	0.007
Secondary	49.5 (7.96)		43.9 (5.03)		43.1 (5.45)	
Tertiary	52.8 (5.87)		45.2 (5.05)		45.5 (4.61)	
**Occupation**						
Private	49.7 (7.85)	0.108	44.4 (5.14)	0.334	44.3 (5.44)	0.007
Government	52.7 (6.45)		44.6 (5.13)		44.5 (4.38)	
Retiree	48.6 (8.18)		43.1 (5.03)		42.2 (5.42)	
Housewives	49.5 (8.51)		44.3 (5.02)		43.2 (5.24)	
Students	51.9 (6.05)		43.4 (4.86)		47.3 (3.59)	
Unemployed	48.7 (7.33)		43.2 (5.91)		43.0 (5.55)	
**Known medical illness**						
Yes	49.7 (7.99)	0.499	43.9 (5.15)	0.317	43.5 (5.35)	0.571
No	48.6 (7.32)		44.9 (4.93)		44.1 (5.23)	
**Area of residence**						
Urban	47.8 (8.25)	< 0.001	42.9 (5.09)	< 0.001	42.6 (5.29)	0.002
Rural	51.1 (7.39)		44.8 (5.06)		44.2 (5.23)	
**Household income**						
Below RM 4360	49.5 (8.00)	0.110	44.0 (5.11)	0.463	43.5 (5.41)	0.316
RM 4360 - RM 9619	50.9 (7.94)		44.8 (5.08)		44.5 (5.08)	
> RM 9619	54.4 (5.06)		44.1 (5.31)		43.1 (5.43)	
**Adherence**						
Good	50.4 (8.02)	0.076	44.6 (4.95)	0.030	44.1 (5.37)	0.116
Moderate and poor	48.6 (7.95)		43.2 (5.23)		42.9 (5.08)	
**Overall (mean score)**	49.6 (7.95)	Range: 23–60	44.0 (5.14)	Range: 30–50	43.6 (5.34)	Range: 26–50
**Overall (in percentage)**	82.6%		88.0%		87.2%	

Notes: Student’s t-test and one-way ANOVA was performed to detect the differences of mean scores across different groups

**Table 8:** Correlation matrix of awareness, expectation and satisfaction (*n* = 441)

Correlations	Awareness scores	Expectation scores	Satisfaction scores
**Awareness scores**	1		
**Expectation scores**	0.462 (*p* < 0.001)	1	
**Satisfaction scores**	0.640 (*p* < 0.001)	0.563 (*p* < 0.001)	1

Notes: Pearson’s correlation was performed to identify the correlation between awareness, expectation and satisfaction scores

## Discussion

To our best knowledge, this was the first local study evaluating patients’ awareness, expectation and satisfaction towards ward pharmacy services. From this study, the respondents demonstrated good awareness towards ward pharmacy service in the medical wards compared to a survey among the inpatients in the United States hospitals [17]. Findings from that study showed poor patients awareness towards the hospital pharmacy services, in which the score was significantly increased after a marketing campaign [17]. In Malaysia, medication history assessment and discharge medication dispensing were among the core services and ‘key performance indicator’ of the ward pharmacy unit [18]. Hence, majority of the respondents were aware about the presence of pharmacists in the ward and their service of medication history assessment and medication dispensing.

Of note, the mean score of patients’ awareness in regard to drug-drug or drug-food interaction and proper storage of medication was lower. Routinely, the ward pharmacists screened for drug-drug or drug-food interactions and discussed with the prescribers prior to dispensing. This process usually occurred without the involvement of patients, which might cause a lower patient’s awareness in this aspect. Nevertheless, our finding was better than a survey among the Ghanaian hospital inpatients, which revealed that only 42% of the patients were provided with information on drug interaction during medication dispensing by pharmacists [19]. On the other hand, proper medication storage was essential to ensure medication safety [20]. The lower score of patients’ awareness about proper medication storage in our study should prompt the ward pharmacists to include these components during medication counseling. These two aforementioned components should be included in routine patient pharmacotherapy education, as comprehensive education may improve patient knowledge and compliance [21–23].

In our study, elderly patients demonstrated significantly lower awareness and satisfaction towards ward pharmacy service. It is noteworthy that elderly patients might have poorer cognitive functions that acted as a barrier in receiving and understanding input provided by the ward pharmacists. Moreover, they demonstrated high reliance on their caregivers to interact with healthcare providers [24]. Therefore, pharmacists interacted more frequently with the caregivers of elderly patients instead of patients themselves during medication history taking and medication counseling. Lack of interaction with the pharmacists due to insufficient time and staffing might contribute to lower awareness and satisfaction among the elderly patients [25]. To improve the elderly patients’ awareness and satisfaction, allocation of more time and the use of ancillary medication counseling tool during pharmacotherapy counseling might benefit the geriatric patients [26].

Our findings suggested that the respondents had high expectation towards the services provided by ward pharmacists. This was comparable to the findings from studies conducted in Ethiopia and Nigeria where the patients had high expectation towards pharmacist to provide professional services and patients’ care [16, 27]. The high score in all of the components in the expectation domain was consistent with the study conducted in a Nigerian teaching hospital, in which patients expected pharmacists to be involved in different aspects of their therapy including prescription screening, medication counseling and monitoring of drugs efficacy [27]. Our result was more encouraging than what was found in a qualitative study among inpatients in a United Kingdom hospital, where a considerable percentage of the patients did not foresee to have interaction with a clinical pharmacist, and did not expect pharmacist to offer clinical input in patient care [15].

Among all, the respondents had high expectation for pharmacist to interact with language that was easily understandable and to explain the function of each medication. This was similar to a study conducted in an Ethiopian hospital pharmacy [16]. Besides that, high expectation was demonstrated in patients with tertiary education. This finding was in congruence with a study by DeWan et al. [28]. Patients with higher education level were likely to have more experience and information, thus this group of patients may have a greater level of expectation [29].

In general, the respondents were highly satisfied towards the ward pharmacy services. This result was similar to another study in Canada which reported 83% participants were highly satisfied on their encounter with inpatient pharmacist and reported a mean satisfaction score of 4.4 out of 5 [30]. Arguably, patients’ satisfaction towards the ward pharmacy services might improve their medication adherence and disease control within a more specific context.

Almost 96% of the respondents were satisfied with the language used by the ward pharmacists, compared to only 68% reported in an Oman study by Jose et al. [13]. The pharmacists employed in Malaysia were usually able to converse in multiple languages including the native language. Conversely in Oman, majority of the pharmacists were expatriates of non-Arabic origin. Previous study also revealed that the use of native language among inpatients had significantly improved patients’ knowledge towards their medications [31]. Hence, the ability of the ward pharmacists to speak a language in accordance to patients’ preference might contribute to the patients’ high satisfaction.

On a different note, previous studies revealed that those with lower than tertiary education had higher satisfaction towards ward pharmacy services [12, 32]. In contrast, our study found that patients with tertiary education had higher satisfaction than those of lower education. This could be due to those with tertiary education have higher self-perceived health status [33]. These patients tend to engage in more medication-related discussion with the ward pharmacists. Furthermore, the respondents were highly satisfied with the level of knowledge on drug related issues demonstrated by the ward pharmacists in our study. Good knowledge towards drug related issues might foster patients’ confidence towards the professionalism of the ward pharmacists. Apart from improving patients’ satisfaction, this might potentially improve medication taking behavior and treatment outcome. It was worth highlighting that the current population demonstrated a higher level of satisfaction towards the ward pharmacists’ clinical knowledge compared to study conducted in Qatar, which reported lower satisfaction level of only 56% [13].

This study had several strengths. Firstly, we achieved a satisfactory response rate of 83.8%, compared to study in similar setting conducted by King et al. which had a response rate of 47% [17]. To our best knowledge, this was the first local study which reported the awareness, expectation and satisfaction of inpatients in the medical ward towards ward pharmacy services. The result of this study could serve as a benchmark in guiding the planning of health policy to improve ward pharmacy services in hospitals.

Our study had its limitations. Although Malaysia was a multiracial country with Malay as the national language and English as a second language, there was still minority of population who could not understand both languages. By excluding this population, in our study, some selection bias might be present. While this was a multicentre study, it was conducted only in the medical wards of Perak state only. Hence, the findings might not reflect entire country and unable to be generalized to other disciplines such as the surgical-orientated wards. Expanding this study to national level and different disciplinary might be useful for a more comprehensive evaluation of awareness, expectation and satisfaction towards various ward pharmacy services in this country.

## Conclusions

Our findings suggested that there was a high level of awareness, expectation and satisfaction towards the ward pharmacy services among patients in the medical wards of public hospitals in Perak state, Malaysia. Older patients have lower awareness towards the role of pharmacists, which warrants training and credentialing of more geriatric pharmacists in addressing geriatric-related medication issues. Awareness on drug storage and drug interaction may be improved via take-home leaflet or digital apps to supplement verbal information provided by the clinical pharmacists.

## Data Availability

The datasets generated and/or analyzed during the current study are not publicly available due to confidentiality of patients, but are available from the corresponding author on reasonable request.
